# Examining the Underappreciated Role of *S*-Acylated Proteins as Critical Regulators of Phagocytosis and Phagosome Maturation in Macrophages

**DOI:** 10.3389/fimmu.2021.659533

**Published:** 2021-04-01

**Authors:** Charneal L. Dixon, Katrina Mekhail, Gregory D. Fairn

**Affiliations:** ^1^ Keenan Research Centre for Biomedical Science, St. Michael’s Hospital, Unity Health Toronto, Toronto, ON, Canada; ^2^ Department of Biochemistry, University of Toronto, Toronto, ON, Canada; ^3^ Department of Surgery, University of Toronto, Toronto, ON, Canada; ^4^ Department of Laboratory Medicine and Pathobiology, Toronto, ON, Canada

**Keywords:** phagocytosis, phagosome, macrophage, palmitoylation, acylation, lipidation

## Abstract

Phagocytosis is a receptor-mediated process used by cells to engulf a wide variety of particulates, including microorganisms and apoptotic cells. Many of the proteins involved in this highly orchestrated process are post-translationally modified with lipids as a means of regulating signal transduction, membrane remodeling, phagosome maturation and other immunomodulatory functions of phagocytes. *S*-acylation, generally referred to as *S*-palmitoylation, is the post-translational attachment of fatty acids to a cysteine residue exposed topologically to the cytosol. This modification is reversible due to the intrinsically labile thioester bond between the lipid and sulfur atom of cysteine, and thus lends itself to a variety of regulatory scenarios. Here we present an overview of a growing number of *S*-acylated proteins known to regulate phagocytosis and phagosome biology in macrophages.

## Introduction

The functionality of many peripheral and membrane proteins depends on the co- or post-translational addition of lipids. Covalent attachment of fatty acids to proteins is one of the more prominent types of lipidation. The type of fatty acylation depends on the fatty acid species and resulting linkage. *N*-terminal glycine myristoylation (*N*-acylation) and cysteine palmitoylation (*S*-acylation) are amongst the most common acylation reactions in mammalian cells; each reaction involves different enzymes, fatty acyl-CoA, and occur in different intracellular locations (1). In either case, the addition of fatty acids markedly increases the hydrophobicity of targeted proteins and modulates function by affecting, binding affinity to biological membranes, subcellular localization and trafficking, folding and stability, and binding interactions with other proteins and co-factors (1, 2).

*S*-palmitoylation is a reversible, post-translational modification of proteins in which palmitic acid (C16:0), is attached to the cysteine residue of a protein substrate *via* a thioester linkage. Nearly all palmitoylated proteins are modified *via* a thioester linkage to cysteine. There are also rare instances of palmitate attached to serine residues (*O*-acylation) and the *N*-terminal of a protein (*N*-acylation) (**Table 1**). The transfer of palmitate from palmitoyl-CoA to the sulfhydryl group of a cysteine residue is catalyzed by a family of protein acyltransferases (PATs) that possess a zinc finger Asp-His-His-Cys (zDHHC) domain (3). Notably, many of the studies cited in this review generally assume that palmitate is the acyl group attached. Although palmitoyl-CoA is the preferred substrate for members of the zDHHC family, other acyl-CoAs such as myristoyl-CoA (C14) and stearoyl-CoA (C18) are also utilized as donors (4); as such a more encompassing and accurate term for this modification is *S*-acylation.

**Table 1:** Fatty acylation of proteins.

Modification	Lipid	Amino acid modified	Linkage
*S*-acylation	C16:0	Cysteine	Thioester (R-SH)
	palmitic acid		
	C18:0		
	stearic acid		
	C16:1		
	palmitoleic acid		
	C18:1		
	oleic acid	
	C20:4		
	arachidonic acid	
*N*-acylation	C14:0	Glycine	Amide (R-NH2)
	myristic acid		
	C16:0		
	palmitic acid	
	C16:0	Cysteine	Amide (R-NH2)
	palmitic acid		
*N*-acylation	C14:0	Lysine	Amide (R-NH2)
	myristic acid		
	C16:0		
	palmitic acid	
*O*-acylation	C16:0	Serine or threonine	Oxyester (R-OH)
	palmitic acid		
	C8:0		
	octanoic acid	
	C16:1		
	palmitoleic acid	

**Figure 1:**
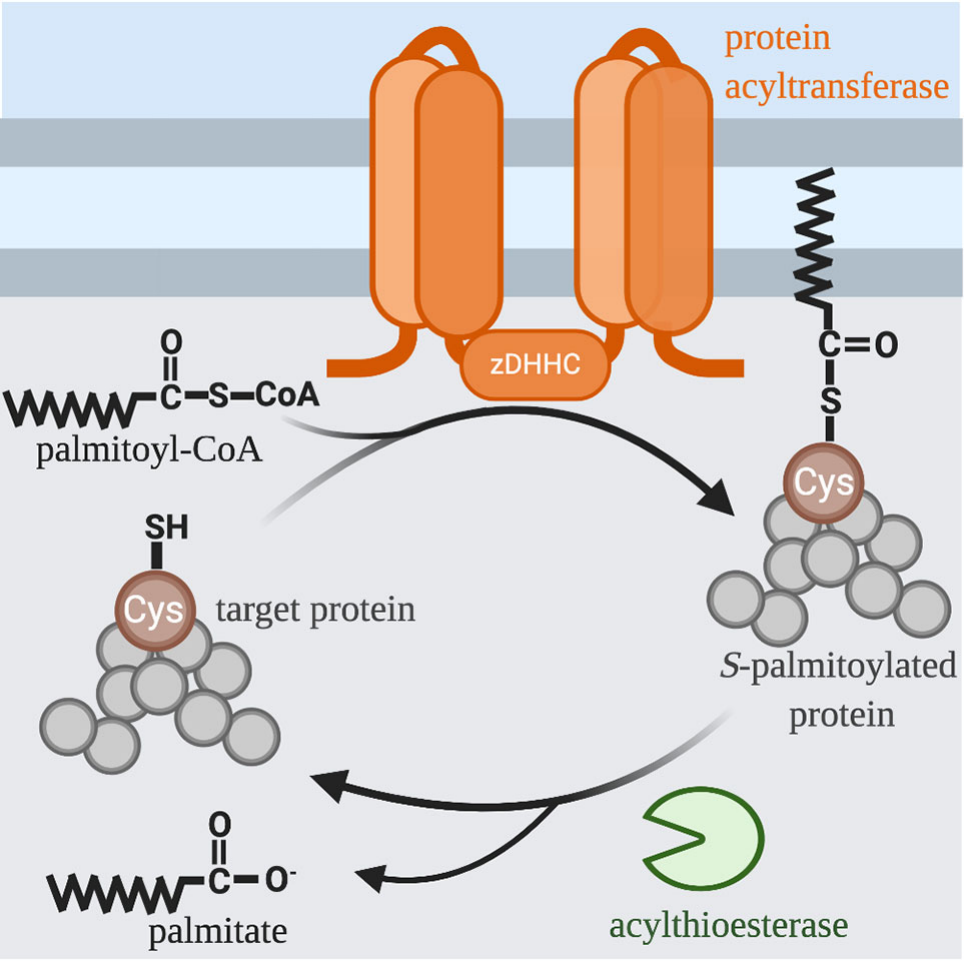
*S*-acylation Cycle. The palmitoyl group is transferred to the free thiol of cysteine from palmitoyl-CoA by a family of integral membrane zinc-dependent DHHC protein acyltransferases. The hydrophobicity of palmitate allows modified proteins to associate with the membranes of various organelles and facilitates trafficking between these organelles. Acyl-protein thioesterases remove thioester-linked fatty acids and thus regenerate a free thiol group.

In contrast to other types of acylation, *S*-acylation is a highly reversible post-translational modification **(** **Figure 1**). Due to the labile nature of the thioester bond, *S*-acylated proteins can undergo a cycle of acylation and deacylation mediated by the opposing activities of PATs and thioesterases, respectively. There are at least 3 classes of enzymes capable of de-acylation: cytosolic acyl protein thioesterases (APT1 and APT2) (5); α/β hydrolase domain-containing (ABHD) proteins (6, 7); and palmitoyl-protein thioesterase 1 (PPT1) that remove palmitate from proteins in the lysosome prior to their degradation (8). The extent to which *S*-acylation is regulated by enzymatic de-acylation is unknown. Still, it is clear that within the cellular context, the cycle of acylation and de-acylation provides an important mechanism for dynamically regulating the localization and function of proteins within a cell. In particular, cell signaling, and membrane trafficking require that secreted proteins are able to reversibly associate with membranes. In this review, we focus on *S*-acylated proteins as regulators of phagocytosis and phagosome maturation.

## Phagocytosis

Phagocytosis is a critical component of both the innate and adaptive immune responses to microbial pathogens (9). It is a specialized form of endocytosis that involves receptor-mediated recognition and ingestion of particles larger than 0.5μm into plasma membrane derived vacuoles called phagosomes. Most cell types are capable of phagocytosis, however only a few immune cells, namely macrophages, neutrophils and dendritic cells are adept at it. These professional phagocytes (10) play a critical role in innate immunity – the first line of defense against infection – by eliminating microorganisms. In addition to their roles in the innate immune response, phagocytes contribute to tissue homeostasis and remodeling by removing apoptotic bodies (11, 12).

**Figure 2:**
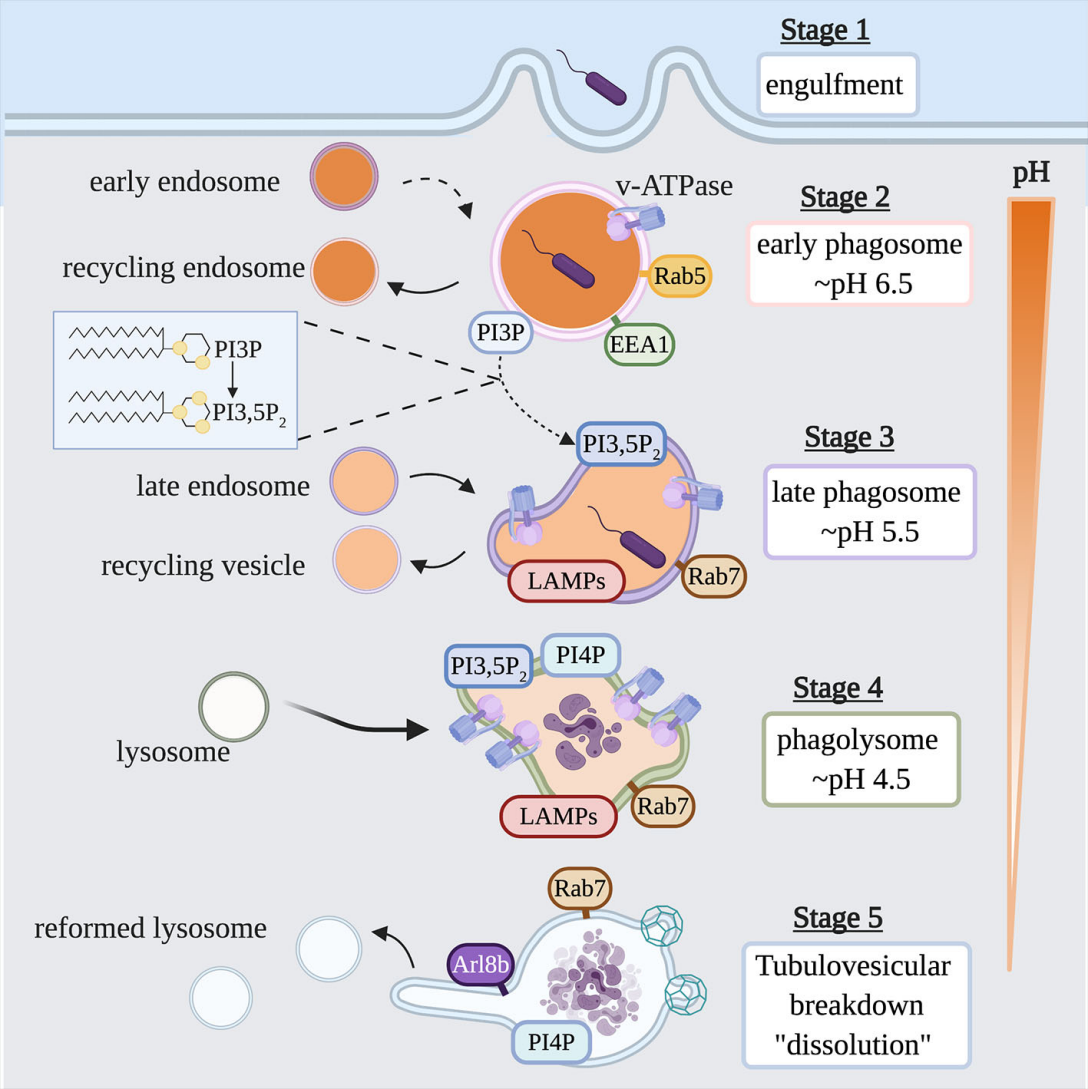
Life Cycle of the Phagosome. Phagosomes are formed on demand when professional phagocytes encounter target particles such as, opsonized bacteria or apoptotic cells. Following internalization of a phagocytic target, the nascent phagosome is transformed into a microbiocidal vacuole through successive fusion and fission events within the endocytic pathway. During this maturation process, the lumen of the phagosome is acidified by the action of v-ATPase, and also acquires hydrolases and other antimicrobial proteins. Key steps in the maturation process include the conversion from a Rab5-positive early phagosome to a Rab7-positive late phagosome; the acquisition of LAMP and v-ATPase; the conversion of PI3P to PI3,5P_2_; and the appearance of PI4P. Once the particle within the phagolysosome has been degraded, PI4P- and Arl8b-dependent membrane tubulation and budding events breakdown the organelle, reforming lysosomes consumed in the maturation process. EEA1, early endosomal antigen 1; LAMP, lysosome-associated membrane protein; PI3P, phosphatidylinositol 3-phosphate; PI3,5P_2_, phosphatidylinositol 3,5- bisphosphate; PI4P, phosphatidylinositol 4-phosphate; Arl8b, ADP-ribosylation factor-like protein 8B.

Phagocytosis is initiated when a plasma membrane bound receptor engages its cognate ligand on the surface of a particle. Receptor engagement initiates complex signaling cascades that lead to particle internalization by inducing localized membrane remodeling of the plasma membrane, and the formation of actin-driven pseudopods which engulf the target particle to form a nascent phagosome (13). The nascent phagosome then follows a highly choreographed pathway of successive fusion and fission events with early endosomes, late endosomes and finally lysosomes, whereby it matures into a microbiocidal vacuole called a phagolysosome (**Figure 2**) (14, 15). At the conclusion of maturation, the lumen of the mature phagolysosome is a highly oxidative environment, rich in hydrolytic enzymes that function at a low pH to destroy the internalized particle. Once the prey within the phagolysosome has been degraded, a tubulovesicular process mediates the dissolution of the vacuole to reform lysosomes which support subsequent rounds of phagocytosis.

The importance of *S*-acylation in cellular processes like phagocytosis has only recently been recognized, owing in large part to the development of methods that allow high throughput analysis of *S*-acylated proteins. The first acyl-biotin exchange proteomic analysis of RAW 264.7 macrophage-like cells identified ~80 *S*-acylated proteins, nearly half of which partition into detergent resistant membrane (DRM) fractions (16). Many of the proteins identified have known roles in signal transduction (Src family kinases (SFKs)) and vesicular transport (soluble NSF attachment proteins (SNAP) receptor (SNARE) proteins (e.g., VAMP3, 4, 5, 7 and Syntaxin6, 7, 8, 12). The identification of these specific proteins highlights the potential importance of *S*-acylation for signaling, optimal completion of particle internalization during phagocytosis, as well as the overall ability of macrophages to perform their functions as sentinels of the immune system.

In addition to the elimination of microorganisms and effete cells *via* phagocytosis, macrophages must also sense and respond to bacterial products such as lipopolysaccharide (LPS) or peptidoglycan (PGN) and mount an effective inflammatory response. Analysis of macrophages, using metabolic labeling with a lipid analog, revealed that when the cells are stimulation with LPS, the *S*-acylated proteome is extensively remodeled, with 154 proteins upregulated and 186 downregulated (17). This study further demonstrated that the cellular pool of phosphatidylinositol 4-kinase IIβ (PI4KIIβ) displays enhanced levels of *S*-acylation following the addition of LPS, and that this lipid kinase is required for the proper production of inflammatory cytokines. The inducible *S-*acylation of the cytosolic PGN sensors NOD1 and NOD2 was recently revealed as a novel regulatory mechanism. Treating primary bone marrow derived macrophage cells with PGN resulted in a zDHHC5-dependent increase in *S*-acylation of NOD1 and NOD2 required for their localization to the plasma membrane or phagosomes and to initiate a signal transduction cascade and NF-kB activation (18). Taken together, these examples demonstrate that dynamic control of protein *S-*acylation in macrophages, and likely most cell types, is a key regulatory mechanism.

### *S*-Acylation of Immune Receptors and SFKs

**Figure 3:**
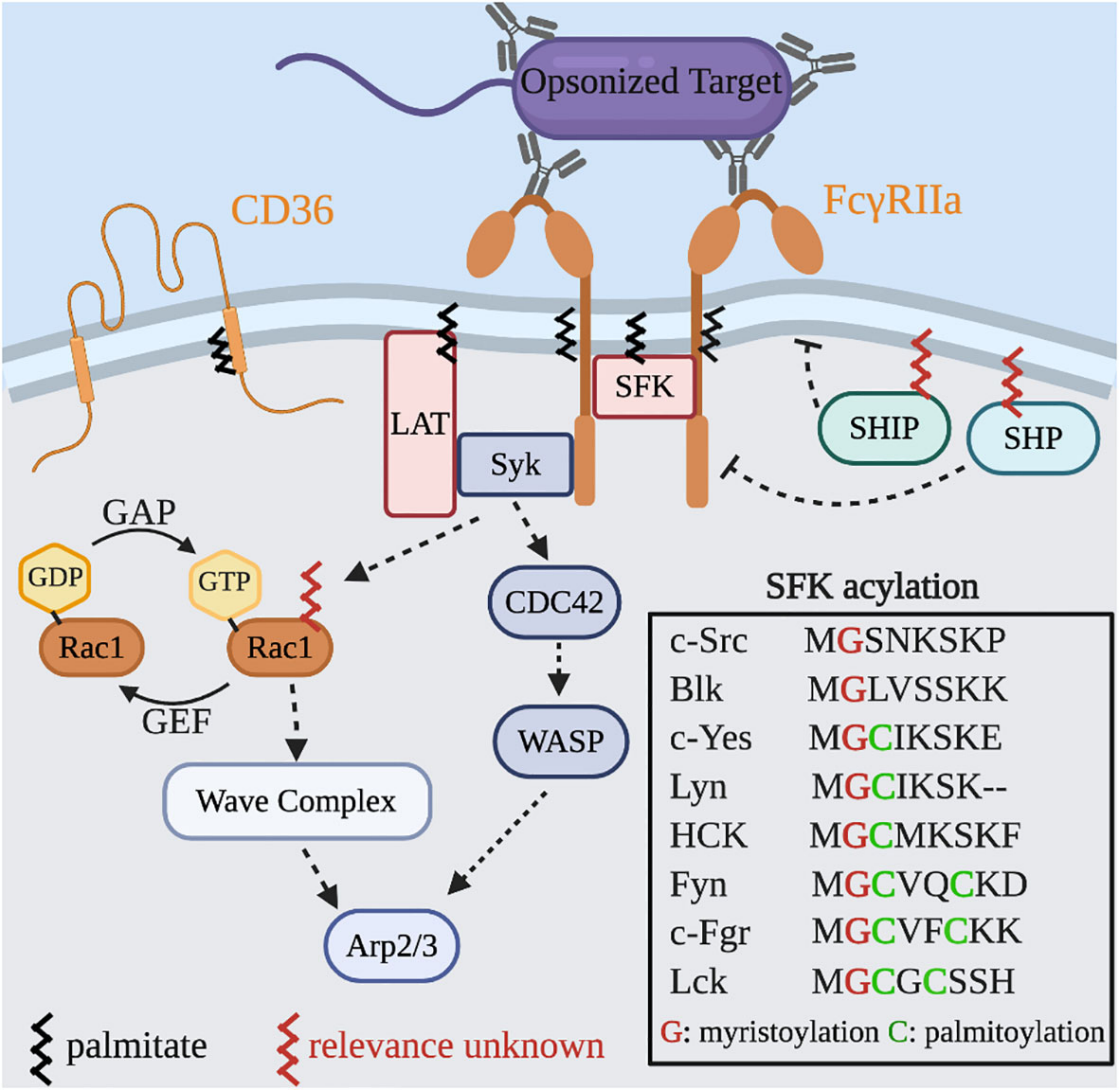
*S*-acylated proteins in Fcγ receptor signaling and actin dynamics. Binding of IgG to the FcγR leads to localized clustering and signal transduction. Phosphorylation of the ITAM motifs and SFKs generates binding sites for Syk and the recruitment of LAT which together amplify the signal intensity. Additional downstream signaling leads to GTP-loading of both Cdc42 and Rac1 that results in Wiskott–Aldrich syndrome protein (WASP) and WASP-family verprolin homologous protein (WAVE) complex mediated actin rearrangements to drive particle engulfment. Two phosphatases, SHP and SHIP, serve as negative regulators of phagocytosis and are recruited by binding the inhibitor FcγRIIb and may also require *S*-acylation for their activity. Inset: All SFKs are myristoylated and most are S-acylated.

Internalization of immunoglobulin G (IgG) opsonized particles by Fcγ receptors (FcγRs) on the surface of macrophages is the most widely used model of phagocytosis. FcγRs bind to bivalent or multivalent ligands to elicit downstream signaling (13). Recognition and binding of complementary IgG on the surface of a particle results in receptor clustering, which brings the cytosolic domain of each FcγR into close apposition and triggers signaling cascades. The first signaling event to occur after receptor-ligand binding is phosphorylation of tyrosine residues within conserved immunoreceptor tyrosine-based activation motifs (ITAMs) on the cytosolic tail of the FcγRs, mediated by Src family kinases (SFKs) (**Figure 3**). Inhibition of SFKs or overexpression of their negative regulator, C-terminal Src kinase (Csk), eliminates particle engulfment, but not binding (19–21). Conversely, the heterologous expression of FcγRs in many non-phagocytic cells is able to elicit SFK signaling cascade and the engulfment of IgG-opsonized particles highlighting the robustness and this system.

Many immunoreceptors including FcγRs are found to reside in DRMs that also contain SFKs (22). The amino acid constituents of transmembrane domains and the *S-*acylation of juxtamembrane regions can partition proteins into liquid ordered regions of the plasma membrane (23). The current evidence suggests that FcγRIIa signaling is only partially dependent on *S-*acylation of Cys208; its absence prevents the intracellular mobilization of calcium without directly impairing the phosphorylation of the ITAMs (24). Additionally, mutations in the transmembrane domain, which ablate its localization to DRMs, prevent the activation of NF-kB downstream of FcγRIIa activation (25). Together these studies demonstrate that both the *S*-acylation and the composition of the transmembrane domain support proper receptor function.

It is possible that multiple activated FcγRs and SFKs associate in an ordered domain to support maximal signal output, and that this depends on the membrane lipids, the transmembrane domain of the receptor, and the recruitment of SFKs. We suspect that under suboptimal conditions the size of this domain could be restricted thereby limiting the overall strength and duration of signals. Similar observations have been seen with another phagocytic receptor, the scavenger receptor CD36 (26). In resting cells, CD36 resides in small clusters associated with the SFK Fyn. Upon activation the clusters increase in size recruiting more Fyn, leading to stronger signaling. Since CD36 is *S-*acylated by zDHHC4 and zDHHC5 (27), and Fyn is dually acylated with myristate and palmitate, it is possible that these modifications assist in lateral segregation and cluster density. Whether this mechanism explains the observations of FcγRIIa remains to be directly tested.

### *S*-Acylated Proteins That Regulate Actin Dynamics and Pseudopod Extension

The role of these initial signaling events is to drive particle internalization through extensive remodeling of the actin cytoskeleton. In FcγR mediated phagocytosis, the small Rho-family GTPases Cdc42 and Rac, key regulators of actin dynamics, are activated and recruited to the site of phagocytosis (28, 29). GTPases function as molecular switches alternating between an active (GTP-bound) state and an inactive (GDP-bound) state (30), through the action of guanine nucleotide exchange factors (GEFs) and GTPase-activating proteins (GAPs), respectively. GEFs stimulate GDP dissociation to allow its replacement by GTP, while GAPs are essential to prompt GTP hydrolysis. The eventual outcome of cytoskeletal reorganization is the formation of pseudopod extensions and phagocytic cups that engulf the particle. Soon after sealing the phagocytic cup, the newly formed phagosome participates in successive fission and fusion interactions with endosomes and lysosomes to mature into a highly degradative phagolysosome.

Rac1 stimulates actin polymerization to promote the internalization of attached particles and microorganisms during phagocytosis (31). In its active GTP-bound state, Rac1 is targeted to membranes by prenylation (geranylgeranylation) and a *C*-terminal polybasic region (KKRKRK) (32, 33). Metabolic labeling in fibroblasts with tritiated palmitate revealed that Rac1 is modified on Cys178 (34, 35). This modification requires Rac1 to already be localized to the membrane as both prenylation and an intact *C*-terminal polybasic region was required to support *S*-acylation. Preventing the *S*-acylation of Rac1 by treatment with 2-bromopalmitate or mutation of the Cys178 residue results in a reduction in both plasma membrane localization and GTP loading of Rac1, which leads to a reduced activation of p21 activated kinase. Ultimately, cells with impaired Rac1 acylation display impaired spreading and defects in migration suggesting defects in the ability to polymerize actin. Whether the *S*-acylation of Rac1 occurs in macrophage or is important for pseudopod extension during phagocytosis is currently unknown. The identity of the of the zDHHC isoform(s) modifying Rac1 also needs to be determined.

### *S*-Acylated Membrane Fusion Machinery Required for Focal Exocytosis During Particle Engulfment

Video-microscopy of phagocytosing macrophages has revealed that these cells have the ability to consume large volumes of particles without a noticeable diminution of their surface area or alteration in shape. Indeed, through the use of fluorescent spectroscopic and electrophysiological techniques (e.g., membrane capacitance) it was determined that the surface area of the cell actually increases immediately following phagocytosis (36, 37). Endocytic vesicles serve as the primary donor of this “extra” membrane used to support particle engulfment (38). This localized delivery at the site of phagocytosis is referred to as “focal exocytosis” (39). Fusion of endocytic vesicles with the base of the phagocytic cup is mediated by the soluble *N*-ethylmaleimide–sensitive factor attachment protein (SNAP) receptors (SNAREs). SNAREs are classified as Qa‐, Qb‐, Qc‐, Qbc‐ (which contain two SNARE motifs) or R‐SNAREs (40, 41). Specific Q‐SNAREs in the target membrane bind, *via* their SNARE motifs, to a partner R‐SNARE on vesicles to form a trans‐SNARE complex (42). This brings the two membranes into close proximity allowing fusion and delivery of cargo. On the plasma membrane and base of the phagocytic cup SNAP-23 (a Qbc) is in complex with a Qa SNARE such as Syntaxin2, 3 or 4. Unlike the Qa SNAREs, SNAP-23 does not have a transmembrane and instead associates with cellular membranes through acylation of a cysteine-rich domain present in the linker region connecting its Qb and Qc motifs (43, 44). SNAP-23 has five acylated cysteines and is found to partition into DRMs (45) which may also explain its preference for the plasma membrane and endosomes over the endoplasmic reticulum. Silencing of SNAP-23 in J774 macrophage cells results in impaired phagocytosis and a reduction in the delivery of NADPH oxidase 2 complex required to generate superoxide at the site of phagocytosis/nascent phagosome (46).

During phagocytosis recycling endosomes, early endosomes and late endosomes/phagosomes can all fuse at the base of the phagocytic cup. The R-SNAREs vesicle-associated membrane protein (VAMP) 3 (recycling endosomes) and VAMP7 (late endosomes/lysosomes) have been shown to be important for membrane fusion. VAMP3 has been identified in proteomics screens as an *S*-acylated protein in macrophages (16, 17). VAMP3 contains a sole cysteine residue, Cys76, located in the cytosol and adjacent to its transmembrane domain. To our knowledge direct examination of this acylation and any potential role has not been examined. Proteomic studies have also identified VAMP7 as an *S*-acylated protein in macrophages (17). Recent studies using Jurkat T cells have demonstrated that Cys183 can be acylated by zDHHC18 and zDHHC20 and that the fraction of VAMP7 that is acylated increases following T cell receptor stimulation (47). In T cells wild-type VAMP7 has a perinuclear localization which partly overlaps with the Golgi marker Giantin and possibly recycling endosomes. Conversely, the Cys183Ala mutant displays a more diffuse cytoplasmic signal demonstrating that *S*-acylation is critical for the proper subcellular distribution. To date the importance of *S*-acylation in macrophage or to phagocytosis has not been examined. However, given the similarity between T cell receptor and FcγR signaling and the role of VAMP7 in phagocytosis it is tempting to speculate that this modification will also support the process.

## Phagosome Maturation

**Figure 4:**
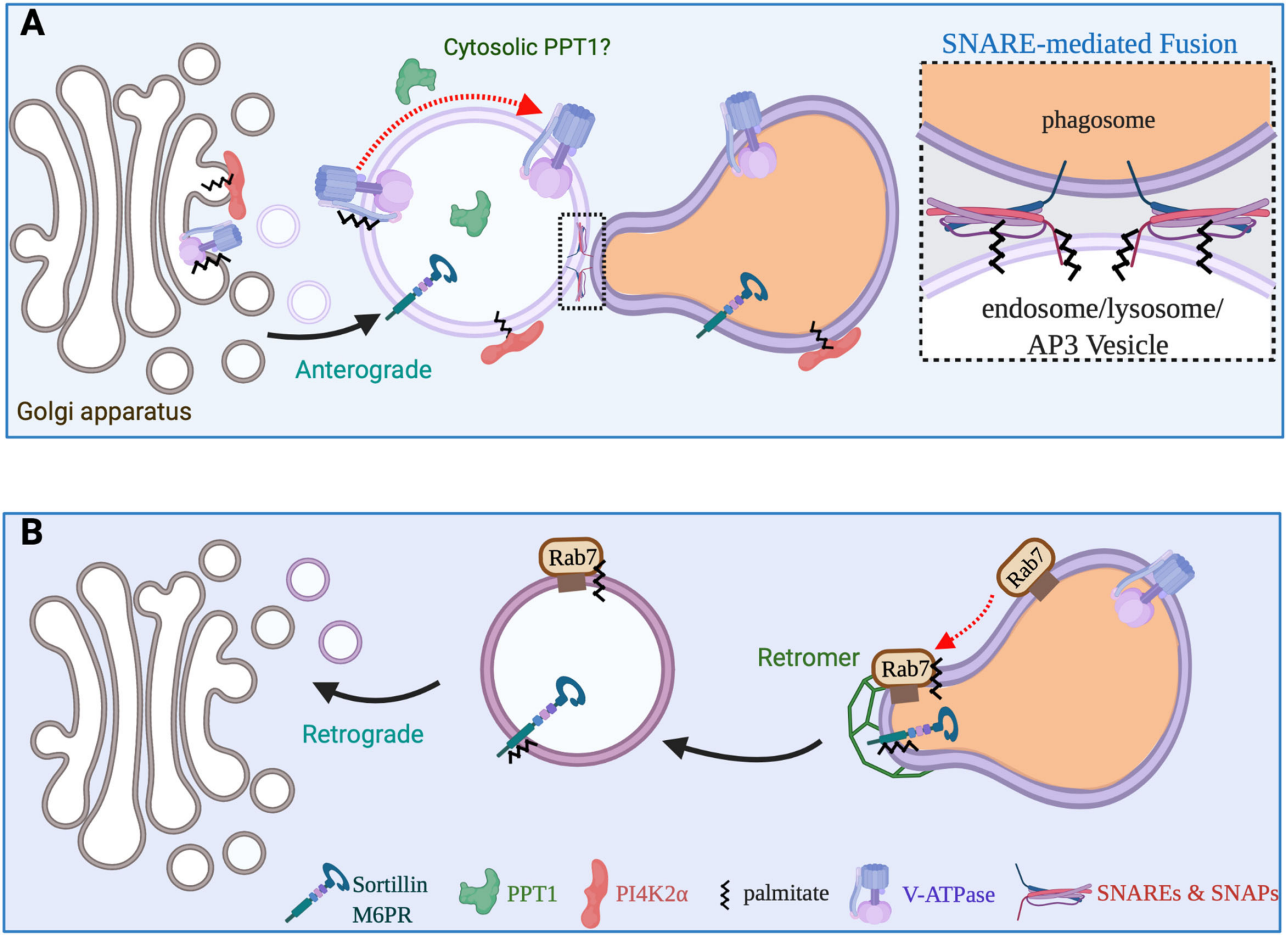
Vesicular transport pathways required for phagosome maturation. **(A)** The maturing phagosome undergoes numerous SNARE-mediated fusion events during the course of its maturation. SNAP23 and SNAP25 are *S*-acylated, while a subset of SNARE proteins also require acylation for proper function and stability. Newly synthesized v-ATPase transits from the Golgi to late endosomes/lysosomes or directly to maturing phagosomes. The clathrin adaptor complex 3 aids in the sorting and delivery of a variety of proteins including the PI4KIIα and cargo receptors such as sortillin to the (phago)lysosome. *S*-acylation of the PI4KIIα and v-ATPase is critical to deliver these enzymes to (phago)lyosomes. **(B)** Cargo receptors M6PR and sortillin must constantly traffic back and forth between the Golgi apparatus and (phago)lysosomes to ensure that pro-hydrolytic enzymes are properly delivered to the lumen of (phago)lysosomes. Both the cation independent M6PR and Sortillin can be *S*-acylated and modification aids in interactions with the retromer complex. Additionally, *S*-acylation of Rab7 also promotes its interactions with the retromer complex and the retrieval cargo receptors.

The nascent phagosome lacks the microbicidal and degradative capacity required to destroy an engulfed particle. Through a sequential process known as maturation, it amasses an arsenal of oxidative, acidifying and hydrolytic enzymes (14, 48). Phagosome maturation, particularly the process of phagolysosome formation, shares some features with the progression of endosomes to lysosomes, a complex process that is orchestrated by Rab GTPases. Soon after sealing the phagocytic cup, the newly formed phagosome fuses with early endosomes and acquires Rab5 and phosphatidylinositol 3-phosphate. Eventually, the early phagosome transitions to a late phagosome that is defined by the acquisition of distinct biochemical markers, such as Rab7; Rab7 acquisition is concomitant with the loss of early markers such as Rab5 (49). Active GTP-bound Rab7 recruits Rab-interacting lysosomal protein (RILP) to the maturing phagosome and, together, they regulate the assembly and activation of the vacuolar ATPase (v-ATPase) leading to further luminal acidification (50, 51). As the phagosome matures it also receives newly synthesized pro-enzymes from the Golgi. Many of these pro-enzymes are packaged in the vesicles arising from the *trans*-Golgi network (TGN) *via* cargo receptors such as mannose 6-phosphate receptor (M6PR) and sortillin (**Figure 4**) (52, 53). These receptors must be actively retrieved from the late phagosome and returned to the Golgi for subsequent rounds of cargo delivery or will otherwise be degraded and the pro-enzymes secreted. The retrograde transport of material from the late phagosomes to the Golgi is incompletely understood but likely involves clathrin machinery and the retromer complex and is possibly regulated by Rab7 (52, 54).

### *S*-Acylated SNAREs Involved in Phagosome Maturation

In addition to supporting focal exocytosis SNAP23 is also critical for mediating membrane fusion events during phagosome maturation. The activity of SNAP23 is necessary for the for the recruitment of several proteins essential for phagosome maturation including v-ATPase (46), MHCI (55) and NOX2 (46, 56, 57) from recycling endosomes and lysosomes. Focal exocytosis of recycling and late endosomes during particle engulfment results in the delivery of a variety of membrane proteins found on these compartments including Syntaxin7 and 13 (also referred to as Syntaxin12) (58). This delivery of endocytic Q-SNAREs to the nascent phagosome allows for further fusions events with recycling and late endosomes/lysosomes containing the appropriate R-SNAREs.

In RAW macrophage, Syntaxin13 colocalizes with the transferrin receptor on recycling endosomes (58). Expression of the cytosolic domain of Syntaxin13, which is known to act as a dominant negative mutant, did not have any impact of the uptake of particles. However, this dominant negative construct impaired the ability of the phagosome to mature to a late phagosome/phagolysosome stage. Syntaxin13 is not reported to be *S*-acylated and it does not contain any juxtamembrane cysteine residues. However, its cognate partner Syntaxin4 has been found to be *S*-acylated (59). The presence of Syntaxin7 and 8 on maturing phagosomes supports the fusion with late endosomes and lysosomes *via* interactions with their cognate pattern VAMP7. Syntaxin7 and 8 are modified with palmitate on Cys239 and Cys214, respectively, adjacent to their transmembrane domains (60). In HeLa cells the Syntaxin 8 is partly colocalized with both CD63 and LAMP1 consistent with a late endosome/lysosome distribution. The Cys214Ala mutant that lacks *S*-acylation has the same subcellular localization. In contrast in HeLa cells Syntaxin7 localizes primarily with the early endosomal marker EEA1 while the Cys239Ala mutant is retained on the plasma membrane of HeLa. This suggests that *S*-acylation is needed for partitioning into an endocytic carrier. Whether this mode of regulation is important in macrophage is yet to be examined.

### *S*-Acylation Regulates Delivery of v-ATPase to Lysosomes and Possibly Phagosomes

Fusion with late endosomes, lysosomes and carriers derived from the TGN lead to the accumulation of v-ATPases in the membrane of the late phagosome that results in the acidification of the phagosomal lumen. Fusion of the maturing phagosome with these compartments also delivers a variety of hydrolytic enzymes to the lumen of the phagolysosome to facilitate destruction of the internalized prey. The v-ATPase is a large multimeric protein complex consisting of two functional subcomplexes, V_1_ and V_0_, that together control the import of protons into the lumen of endosomes and phagosomes (61). The cytosolic V_1_ subcomplex mediates ATP hydrolysis, while the V_0_ subcomplex, integral to the membrane, constitutes the pore through which protons are pumped at the expense of ATP (62). The phagosomal acquisition of the membrane embedded V_0_ subcomplex requires membrane trafficking either from pre-existing active complex (i.e., fusion with lysosomes) or the delivery of newly synthesized V_0_ from the ER *via* the Golgi (**Figure 4**).

Batten disease is a group of rare, fatal, genetically inherited disorders that impact the nervous system which all manifest as neuronal ceroid lipofuscinoses (63). Infantile Batten disease, or ceroid lipofuscinosis, neuronal, 1 (*CLN1*) is a severe form of the disease caused by mutations in the thioesterase PPT1 (64). PPT1 is a soluble protein thioesterase enzyme that resides in the lumen of lysosomes involved in the degradation of lipid-modified proteins by removing thioester-linked fatty acyl groups from cysteine residues (65). Delivery of recombinant PPT1 *via* fluid phase endocytosis is able to reverse the accumulation of proteinaceous deposits in the lysosomes lacking PPT1 function. Paradoxically, recent evidence suggests that the activity of the v-ATPase also requires PPT1 to directly remove an acyl chain from the v-ATPase in the cytosol.

Neurons, macrophage and microglia express the V_0_a1 isoform as part of the membrane embedded V_0_ complex of the v-ATPase. A recent study has demonstrated that the V_0_a1 containing Vo complex requires S-acylation for proper trafficking from the Golgi to lysosomes (66). This feature is unique to the a1 isoform compared to the other tissue-selective isoforms (a2-a4). Cys25 of the a1 subunit was shown to be *S-*acylated by acyl-biotin exchange and that this modification is important for the recognition of the V_0_ complex by the adaptor complex 3 in the *trans*-Golgi and its subsequent delivery to lysosomes. Curiously, despite its role in trafficking, the modification itself impairs maximal proton translocation activity (66). Mutations in PPT1 result in a significantly higher lysosomal pH in neurons compared to those from littermate controls. It is currently unclear how lysosomal PPT1 can remove fatty acids attached to a cytosolically modified Cys residue. One possibility is that a small fraction of the PPT1 also resides in the cytosol (66). Whether this is through inefficient insertion into the ER during biosynthesis or escape *via* some sort of transient “leak” in limiting membrane of the lysosome is unclear. Since some bacterial pathogens and inert particles can rupture phagolysosomes it would be worth investigating to determine if the release of PPT1 into the cytosol impacts the *S*-acylome of macrophages.

### *S*-Acylation of PI4KIIα and Its Delivery and Activity Are Required for Optimal Phagolysosome Acidification

Phosphatidylinositol 4-kinase IIa (PI4KIIα) resides primarily in the *trans*-Golgi network (TGN) and endosomes/lysosomes and plays essential roles in membrane transport including: cargo trafficking between the TGN and endosomes *via* direct interactions with adaptor protein complexes, AP-1 and AP-3 (67–69). Importantly both the activity and the presence of PI4KIIα supports the formation of vesicular carriers (68). PI4KIIα is a peripheral membrane protein that is *S-*acylated within its catalytic core (70). All four cysteine residues within its CCPCC motif are capable of being *S*-acylated by zDHHC3 and/or zDHHC7 (71). However, mutational analysis indicates that the first two are the preferred sites of this modification (70).

Recently it was also demonstrated that following the acquisition of Rab7, the maturing late phagosome, acquires PI4KIIα by fusion with lysosomes or vesicles from the TGN. This leads to the appearance of PI4P on this late stage phagosome and is required for the maximal acidification of the now phagolysosome (72). Additionally, in dendritic cells PI4K2a and PI4P have been shown to be critical for the localization and activity of toll-like receptor 4 (73). Following the digestion of the prey within the phagolysosome, the organelle is eliminated by a tubulovesicular dissolution process, which concomitantly regenerates lysosomes. The precise mechanisms of this phagolysosomal dissolution or resolution are unclear, however, it is known that if the phagolysosome fails to acquire PI4P its dissolution in inhibited (74). Moreover, similar PI4P/PI4K2a positive tubules in dendritic cells may account for the requirement for PI4K2a to support optimal presentation of antigens by the major histocompatibility complex II (73). Thus, we would predict that loss of both zDHHC3 and zDHHC7 would result in an inability of phagolysosomes to degrade prey and be broken down into lysosomes.

### A Potential Role for *S*-Acylation of Rab7 in Retrograde Transport From the Phagolysosome to Trans-Golgi Network

In non-myeloid cells, Rab7 is *S*-acylated on Cys83 and Cys84 and this regulates the ability of Rab7 to interact with and recruit the retromer complex to late endosomes (75). Prevention of Rab7 acylation results in the aberrant secretion of lysosomal pro-enzyme to the extracellular medium. Although the *S*-acylation of these two residues has now been demonstrated, it is still not fully understood how this regulates the retromer. While the role of Rab7 acylation has not been investigated in phagosome maturation, it is tempting to speculate that it will also be required for retrograde transport of cargo receptors such as the mannose 6-phosphate receptor or sortillin back to the Golgi. In a separate investigation identified zDHHC15 as a protein required for optimal retrograde lysosome to Golgi transport of sortillin and the cation-independent M6PR (76). This study also demonstrated that acylation was required for these two cargo proteins to associate with the retromer complex. This raises the possibility that zDHHC15 may directly modify Rab7 to mediate retrograde transport from lysosomes/phagosomes to the *trans*-Golgi Network.

## Why Do Macrophages That Lack zDHHC5 Eat Faster?

Using the human myeloid cell line U937, a recent CRISPR screen identified numerous genes that either positively or negatively regulated phagocytosis of numerous phagocytic prey (77). The authors demonstrated that loss of zDHHC5 resulted in a substantial increase in phagocytosis mediated by a variety of receptors including FcγR, complement, dectin and phosphatidylserine (apoptotic body). This finding was substantiated by another study using murine bone marrow derived macrophage cells lacking zDHHC5 (18). Together, these studies reveal that key negative regulator(s) of phagocytosis require zDHHC5-mediated *S*-acylation for their proper function and that in the absence of this post-translational modification, phagocytic signaling is either enhanced and/or accelerated.

In addition to ITAM containing receptors, there exist comparable receptors which contain immunoreceptor tyrosine-based inhibitory motifs (ITIMs). These include FcγIIb and the phosphatidylserine receptor CD300a (78, 79). Two key molecules which associate with the inhibitory ITIM motifs are Src homology 2 (SH2)-containing tyrosine phosphatase (SHP) and SH2 domain containing inositol phosphatase (SHIP) (80) both of which inhibit phagocytosis by directly dephosphorylating phosphotyrosine and phosphatidylinositol 3,4,5-trisphosphate, respectively, essential for phagocytosis. Proteomics screens for *S-*acylated proteins in RAW264.7 macrophage-like cells have identified both SHP (17, 81) and SHIP (81). We speculate that the plasmalemmal localized zDHHC5 acylates one or both of these proteins which is required for their optimal function. In the absence of lipidation, these phosphatases would have impaired targeting to the sites of receptor activation leading to enhanced signaling and faster rates of phagocytosis.

## Conclusions and Perspectives

In this review, we have focused on the role of experimentally validated *S*-acylated proteins as regulators of phagocytosis and phagosome maturation in macrophages. It is clear from these examples that protein acylation can contribute to membrane binding, trafficking and targeting. All of which can be explained, at least in part, by two molecular mechanisms: the hydrophobic insertion of an acyl chain into a lipid bilayer enhances membrane binding, and saturated fatty acids prefer to insert into liquid ordered raft domains rather than the bulk plasma membrane. It is intriguing to speculate whether the function of *S*-acylated proteins is directly associated with the acylation process or is a consequence of correct trafficking or localization of the target protein. *S*-acylation of FcγRIIa and Rac1 exemplify the versatility of *S*-acylation in modifying protein function beyond membrane binding and targeting. In both instances’ acylation is essential for downstream signaling pathways. However, the relationship between *S*-acylation and protein function isn’t always so transparent as we see for many of the proteins reviewed here. As more *S*-acylated proteins and PATs are identified, knockdown, knockout and knockin techniques will complement the biochemical studies to help elucidate the functions of *S*-acylation.

Finally, our collective understanding of the cellular roles of protein *S*-acylation has lagged behind other post-translational modifications such as phosphorylation, ubiquitination and prenylation. Although in the past few years the identification of several palmitoyl transferases as well as their protein substrates has brought major advances to the protein palmitoylation field many central questions remain. Part of the challenge historically has been the limited number of tools, techniques and reagents to study *S*-palmitoylation. However, the development of new methods using bioorthogonal chemical reporters, ABE and the closely related acyl PEG and acyl-RAC assays, have accelerated both proteomic and directed studies. Additionally, the recent cryo-EM structures of zDHHC15 and zDHHC20 will no doubt help bolster the development of better ligands for *in vitro* studies and inhibitors for cell and animal studies. As the field continues to grow, we believe that the macrophage and the process of phagocytosis and phagosome maturation will be excellent paradigms to study the role of zDHHC PATs, thioesterases and their modified substrates.

## Author Contributions 

CD and GF wrote the paper. KM prepared the figures. All authors contributed to the article and approved the submitted version.

## Funding 

This work is supported by the Canadian Institutes of Health Research Project Grants PJT166010 and PJT165968 to GF.

## Conflict of Interest

The authors declare that the research was conducted in the absence of any commercial or financial relationships that could be construed as a potential conflict of interest.
