# Supplementary material for: Examining the Underappreciated Role of S-Acylated Proteins as Critical Regulators of Phagocytosis and Phagosome Maturation in Macrophages
Source: Front Immunol. 2021 Apr 1;12:659533. doi: 10.3389/fimmu.2021.659533 (PMC8047069; doi:10.3389/fimmu.2021.659533)
Supplement: Supplementary file 1 [file Table_1.docx]

Supplementary Table

|  |  |  |  |  |  |
| --- | --- | --- | --- | --- | --- |
| **TABLE S1** | | | | | |
| **Protein** | ***S*-acylation sites** | **zDHHC** | **Thioesterase** | **Role of S-acylation** | **Reference** |
| FcγRIIa | C208 |  |  | Required for receptor signaling integrity and stability in lipid rafts | 4, 24 |
| CD-36 | C3, C7, C464, C466 | DHHC5, DHHC6 |  | Required for ER to plasma membrane trafficking | 28 |
| LAT | C26, C29 | DHHC18 |  | Required for tyrosine phosphorylation and lipid raft targeting | 17, 32 |
| Rab7 | C83, C84 | DHHC1, DHHC8 |  | Required for recruitment of retromer and trafficking of the lysosomal sorting receptors to TGN | 16 |
| v-ATPase | C25 |  | PPT1 | Regulates interaction with adapter AP2 and AP3 | 3 |
| Vamp7 | C183 | DHHC18 |  | Required for Golgi localization | 17 |
| SNAP-23 | C79, C80, C83, C85, C89 | DHHC2, DHHC3 DHHC7, DHHC17 | APT1 | Required for lipid raft targeting | 25, 32 |
| SNAP-25 | C85, C88, C90, C92 | DHHC2, DHHC3 DHHC7, DHHC8  DHHC15, DHHC17 |  | Required for lipid raft targeting and endosome localization | 7, 10, 25 |
| Syntaxin 7 | C239 |  |  | Required for internalization at the plasma membrane | 9 |
| Syntaxin 8 | C214 |  |  | unknown | 9 |
| PI4KII𝛼 | C173, C174, C176, C177 | DHHC3, DHHC7 |  | Required for targeting to TGN and proper (integral) membrane association. | 14 |
| MHCI | C309, C326 |  |  | Required for egress from endoplasmic reticulum to cell surface | 8 |
| Lck | C3, C5 | DHHC2, DHHC17 DHHC18, DHCC21 | APT1 | Required for plasma membrane localization and T-cell signaling | 1, 2, 6, 11, 29, 31 |
| Lyn | C3 |  |  | Required for PM association | 27, 30 |
| Hck | C3 |  |  |  | 23 |
| Fyn | C3, C6 | ZDHHC2, ZDHHC3 ZDHHC7, ZDHHC10 ZDHHC15, ZDHHC20 DHHC21 | PPT1 |  | 12, 15, 26, 27 |
| Yes | C3 |  |  |  | 27 |
| Cdc42 | C189 | DHHC8 |  |  | 19, 22 |
| Rac1 | C178 | ZDHHC3 |  |  | 20 |
| Flotillin-1 | Cys34 | DHHC5 |  |  | 5, 18 |
| Flotillin-2 | Cys4, 19, 20 | DHHC5 |  |  | 13, 21 |
|  |  |  |  |  |  |

**References**

1. Aicart-Ramos, C., Valero, R.A., and Rodriguez-Crespo, I. (2011). Protein palmitoylation and subcellular trafficking. *Biochimica et Biophysica Acta (BBA) - Biomembranes* 1808(12)**,** 2981-2994. doi: 10.1016/j.bbamem.2011.07.009.
2. Akimzhanov, A.M., and Boehning, D. (2015). Rapid and transient palmitoylation of the tyrosine kinase Lck mediates Fas signaling. *Proceedings of the National Academy of Sciences* 112(38)**,** 11876-11880. doi: 10.1073/pnas.1509929112.
3. Bagh, M.B., Peng, S., Chandra, G., Zhang, Z., Singh, S.P., Pattabiraman, N., et al. (2017). Misrouting of v-ATPase subunit V0a1 dysregulates lysosomal acidification in a neurodegenerative lysosomal storage disease model. *Nature Communications* 8(1)**,** 14612. doi: 10.1038/ncomms14612.
4. Barnes, N.C., Powell, M.S., Trist, H.M., Gavin, A.L., Wines, B.D., and Hogarth, P.M. (2006). Raft localisation of FcγRIIa and efficient signaling are dependent on palmitoylation of cysteine 208. *Immunology Letters* 104(1-2)**,** 118-123. doi: 10.1016/j.imlet.2005.11.007.
5. Brent (2013). Chemical approaches for profiling dynamic palmitoylation. *Biochemical Society Transactions* 41(1)**,** 43-49. doi: 10.1042/bst20120271.
6. Fukata, M., Fukata, Y., Adesnik, H., Nicoll, R.A., and Bredt, D.S. (2004). Identification of PSD-95 Palmitoylating Enzymes. *Neuron* 44(6)**,** 987-996. doi: 10.1016/j.neuron.2004.12.005.
7. Greaves, J., Gorleku, O.A., Salaun, C., and Chamberlain, L.H. (2010). Palmitoylation of the SNAP25 Protein Family. *Journal of Biological Chemistry* 285(32)**,** 24629-24638. doi: 10.1074/jbc.m110.119289.
8. Gruda, R., Achdout, H., Stern-Ginossar, N., Gazit, R., Betser-Cohen, G., Manaster, I., et al. (2007). Intracellular Cysteine Residues in the Tail of MHC Class I Proteins Are Crucial for Extracellular Recognition by Leukocyte Ig-Like Receptor 1. *The Journal of Immunology* 179(6)**,** 3655-3661. doi: 10.4049/jimmunol.179.6.3655.
9. He, Y., and Linder, M.E. (2009). Differential palmitoylation of the endosomal SNAREs syntaxin 7 and syntaxin 8. *Journal of Lipid Research* 50(3)**,** 398-404. doi: 10.1194/jlr.m800360-jlr200.
10. Huang, K., Sanders, S., Singaraja, R., Orban, P., Cijsouw, T., Arstikaitis, P., et al. (2009). Neuronal palmitoyl acyl transferases exhibit distinct substrate specificity. *The FASEB Journal* 23(8)**,** 2605-2615. doi: 10.1096/fj.08-127399.
11. Kabouridis, P.S. (1997). S-acylation of LCK protein tyrosine kinase is essential for its signalling function in T lymphocytes. *The EMBO Journal* 16(16)**,** 4983-4998. doi: 10.1093/emboj/16.16.4983.
12. Koster, K.P., and Yoshii, A. (2019). Depalmitoylation by Palmitoyl-Protein Thioesterase 1 in Neuronal Health and Degeneration. *Frontiers in Synaptic Neuroscience* 11. doi: 10.3389/fnsyn.2019.00025.
13. Li, Y., Martin, B.R., Cravatt, B.F., and Hofmann, S.L. (2012). DHHC5 Protein Palmitoylates Flotillin-2 and Is Rapidly Degraded on Induction of Neuronal Differentiation in Cultured Cells. *Journal of Biological Chemistry* 287(1)**,** 523-530. doi: 10.1074/jbc.m111.306183.
14. Lu, D., Sun, H.-Q., Wang, H., Barylko, B., Fukata, Y., Fukata, M., et al. (2012). Phosphatidylinositol 4-Kinase IIα Is Palmitoylated by Golgi-localized Palmitoyltransferases in Cholesterol-dependent Manner. *Journal of Biological Chemistry* 287(26)**,** 21856-21865. doi: 10.1074/jbc.m112.348094.
15. Mill, P., Lee, A.W.S., Fukata, Y., Tsutsumi, R., Fukata, M., Keighren, M., et al. (2009). Palmitoylation Regulates Epidermal Homeostasis and Hair Follicle Differentiation. *PLoS Genetics* 5(11)**,** e1000748. doi: 10.1371/journal.pgen.1000748.
16. Modica, G., Skorobogata, O., Sauvageau, E., Vissa, A., Yip, C.M., Kim, P.K., et al. (2017). Rab7 palmitoylation is required for efficient endosome-to-TGN trafficking. *Journal of Cell Science* 130(15)**,** 2579-2590. doi: 10.1242/jcs.199729.
17. Morrison, E., Wegner, T., Zucchetti, A.E., Álvaro-Benito, M., Zheng, A., Kliche, S., et al. (2020). Dynamic palmitoylation events following T-cell receptor signaling. *Communications Biology* 3(1). doi: 10.1038/s42003-020-1063-5.
18. Morrow, I.C., Rea, S., Martin, S., Prior, I.A., Prohaska, R., Hancock, J.F., et al. (2002). Flotillin-1/Reggie-2 Traffics to Surface Raft Domains via a Novel Golgi-independent Pathway. *Journal of Biological Chemistry* 277(50)**,** 48834-48841. doi: 10.1074/jbc.m209082200.
19. Moutin, E., Nikonenko, I., Stefanelli, T., Wirth, A., Ponimaskin, E., De Roo, M., et al. (2016). Palmitoylation of cdc42 Promotes Spine Stabilization and Rescues Spine Density Deficit in a Mouse Model of 22q11.2 Deletion Syndrome. *Cerebral Cortex*. doi: 10.1093/cercor/bhw183.
20. Navarro-Lérida, I., Sánchez-Perales, S., Calvo, M., Rentero, C., Zheng, Y., Enrich, C., et al. (2012). A palmitoylation switch mechanism regulates Rac1 function and membrane organization. 31(3)**,** 534-551. doi: 10.1038/emboj.2011.446.
21. Neumann-Giesen, C., Falkenbach, B., Beicht, P., Claasen, S., Lüers, G., Stuermer, C.A.O., et al. (2004). Membrane and raft association of reggie-1/flotillin-2: role of myristoylation, palmitoylation and oligomerization and induction of filopodia by overexpression. *Biochemical Journal* 378(2)**,** 509-518. doi: 10.1042/bj20031100.
22. Nishimura, A., and Linder, M.E. (2013). Identification of a Novel Prenyl and Palmitoyl Modification at the CaaX Motif of Cdc42 That Regulates RhoGDI Binding. *Molecular and Cellular Biology* 33(7)**,** 1417-1429. doi: 10.1128/mcb.01398-12.
23. Robbins, S.M., Quintrell, N.A., and Bishop, J.M. (1995). Myristoylation and differential palmitoylation of the HCK protein-tyrosine kinases govern their attachment to membranes and association with caveolae. *Molecular and Cellular Biology* 15(7)**,** 3507-3515. doi: 10.1128/mcb.15.7.3507.
24. Rosales, C., and Uribe-Querol, E. (2017). Phagocytosis: A Fundamental Process in Immunity. *BioMed Research International* 2017**,** 1-18. doi: 10.1155/2017/9042851.
25. Salaün, C., Gould, G.W., and Chamberlain, L.H. (2005). The SNARE Proteins SNAP-25 and SNAP-23 Display Different Affinities for Lipid Rafts in PC12 Cells. *Journal of Biological Chemistry* 280(2)**,** 1236-1240. doi: 10.1074/jbc.m410674200.
26. Sapir, T., Segal, M., Grigoryan, G., Hansson, K.M., James, P., Segal, M., et al. (2019). The Interactome of Palmitoyl-Protein Thioesterase 1 (PPT1) Affects Neuronal Morphology and Function. *Frontiers in Cellular Neuroscience* 13. doi: 10.3389/fncel.2019.00092.
27. Sato, I., Obata, Y., Kasahara, K., Nakayama, Y., Fukumoto, Y., Yamasaki, T., et al. (2009). Differential trafficking of Src, Lyn, Yes and Fyn is specified by the state of palmitoylation in the SH4 domain. *Journal of Cell Science* 122(7)**,** 965-975. doi: 10.1242/jcs.034843.
28. Thorne, R.F., Ralston, K.J., De Bock, C.E., Mhaidat, N.M., Zhang, X.D., Boyd, A.W., et al. (2010). Palmitoylation of CD36/FAT regulates the rate of its post-transcriptional processing in the endoplasmic reticulum. 1803(11)**,** 1298-1307. doi: 10.1016/j.bbamcr.2010.07.002.
29. Tsutsumi, R., Fukata, Y., Noritake, J., Iwanaga, T., Perez, F., and Fukata, M. (2009). Identification of G Protein   Subunit-Palmitoylating Enzyme. 29(2)**,** 435-447. doi: 10.1128/mcb.01144-08.
30. Wang, J., Hao, J.-W., Wang, X., Guo, H., Sun, H.-H., Lai, X.-Y., et al. (2019). DHHC4 and DHHC5 Facilitate Fatty Acid Uptake by Palmitoylating and Targeting CD36 to the Plasma Membrane. *Cell Reports* 26(1)**,** 209-221.e205. doi: 10.1016/j.celrep.2018.12.022.
31. Zeidman, R., Buckland, G., Cebecauer, M., Eissmann, P., Davis, D.M., and Magee, A.I. (2011). DHHC2 is a proteinS-acyltransferase for Lck. *Molecular Membrane Biology* 28(7-8)**,** 473-486. doi: 10.3109/09687688.2011.630682.
32. Zhang, W., Trible, R.P., and Samelson, L.E. (1998). LAT Palmitoylation. *Immunity* 9(2)**,** 239-246. doi: 10.1016/s1074-7613(00)80606-8.
